# Posterior Pericardiotomy and Its Impact on Cardiac Tamponade and Pericardial Effusion after Cardiac Surgery

**DOI:** 10.5761/atcs.oa.25-00075

**Published:** 2025-07-03

**Authors:** Ismail Al-Shameri, Abudar A. Al-Ganadi, Tarq Noman, Mahdi A. Kadry, Ihab M. Elsharkawy, Naseem Al-Wsabi, Ayman A. Mohammed

**Affiliations:** 1 Department of Cardiovascular Surgery, Cardiovascular and Kidney Transplantation Center, Faculty of Medicine, Taiz University, Taiz, Yemen; 2 Department of Cardiovascular Surgery, Faculty of Medicine, Sana’a University, Sana’a, Yemen; 3 Department of Cardiothoracic Surgery, Faculty of Medicine, Cairo University, Cairo, Egypt; 4 Department of Cardiology, Faculty of Medicine, Taiz University, Taiz, Yemen

**Keywords:** pericardial effusion, post cardiac surgery, posterior pericardiotomy, pericardial tamponade

## Abstract

**Purpose:** Pericardial effusion (PE), tamponade, and atrial fibrillation are challenging complications after cardiac surgeries. This prospective randomized study was conducted to evaluate the impact of posterior pericardiotomy (PP) in the prevention of PE and cardiac tamponed after adult cardiac surgery.

**Methods:** This single-center, prospective, randomized controlled trial included 330 patients undergoing open-heart surgery. They were randomly assigned to either a PP group or a control group.

**Results:** Of 703 screened patients, 330 were enrolled from January 2022 to June 2024 (mean age: 50.2 ± 14.7 years, 64.2% males). Compared to controls, the PP group had significantly lower early and late PE (19.4% vs. 44.8%, and 4.2% vs. 17%, respectively), tamponade (2.4% vs. 11.5%), and postoperative atrial fibrillation (10.3% vs. 19.4%). PP also significantly reduced the need for surgical re-exploration, duration of mechanical ventilation, and both intensive care unit and overall hospital stays (all P <0.05). Adjusted multivariate analysis confirmed the benefits of PP after correcting for baseline imbalances in left ventricular ejection fraction and operative time. No adverse events directly attributable to PP were noted.

**Conclusions:** PP is a simple, safe, and effective technique for reducing postoperative PE, and cardiac tamponade after cardiac surgery.

## Abbreviations

PE
: pericardial effusion
POAF
: postoperative atrial fibrillation
PP
: posterior pericardiotomy
POD
: postoperative day
CPB
: cardiopulmonary bypass
CXR
: chest radiograph
ECG
: electrocardiogram
TTE
: transthoracic echocardiography
SD
: standard deviations
CI
: confidence intervals
ICU
: intensive care unit
LOS
: lounge of stay
RCTs
: randomized controlled trials

## Introduction

Postoperative bleeding, pericardial effusion (PE), and arrhythmia—especially atrial fibrillation (AF)—are among the most common and important postoperative complications.^1–4)^ PE is a frequent complication following open-heart surgery, occurring in approximately 53%–85% of cases.^2,5)^ Postoperative PE is typically minimal and gradually reabsorbed. While often benign, it can result in severe and potentially life-threatening complications, such as cardiac tamponade, and is associated with an increased risk of AF.^3,4,6–8)^ AF is a common complication post-cardiac surgery, affecting 10%–65% of patients.^9)^

Following cardiac surgery, patients are typically positioned supine, which can facilitate fluid accumulation in the posterior pericardial cavity. Standard postoperative drainage involves placing one drain in the pleural space and another beneath the sternum. However, these drains may be insufficient for adequately evacuating fluid from the posterior pericardial space. Additionally, placing a drain directly within the pericardial cavity is often unfeasible during coronary artery bypass grafting (CABG), as it may exert pressure on the grafts.^6,10,11)^ Therefore, implementing an effective method for draining the pericardial cavity after open-heart surgery is essential to mitigate or prevent the above-mentioned complications.^12)^

Posterior pericardiotomy (PP) involves creating an opening from the posterior part of the pericardial space into the left pleural space; this is performed by the surgeon at the end of the operation to minimize fluid accumulation within the pericardial space, thereby reducing the incidence of PE and its associated complications.^13)^ The study was conducted at a single institution in Yemen. However, this center serves as a national tertiary referral center, receiving patients from across the country with varied demographic and clinical backgrounds. Therefore, the included population represents diverse patient profiles from multiple regions of Yemen, although regional and institutional variability elsewhere may still limit complete generalizability.

## Methods and Patients

### Study population and study design

This is a prospective randomized study that screened all consecutive patients who underwent CABG, valvular heart, or combined valvular heart and CABG surgeries between January 2022 to June 2024 in the cardiac surgery departments at the Cardiovascular and Kidney Transplant Center in Taizz, Yemen. Each subject signed informed consent; the study was performed considering the Declaration of Helsinki and was approved by the Institutional Review Committee.

The exclusion criteria were (1) age <18 years; (2) patients with hepatic or renal dysfunction; (3) those undergoing re-operative cardiac surgery; (4) surgeries involving the descending thoracic or thoracoabdominal aorta; (5) patients who had preoperative coagulation, or those who did not provide consent for participation in the research were also excluded.

Participants were randomly assigned into 2 equal groups: the PP intervention group (Group I) and the no-intervention control group (Group II). Simple randomization was performed using sealed, opaque envelopes. Echocardiographic outcome assessments were conducted by investigators blinded to group allocation.

Data were prospectively collected from the time of enrollment throughout the entire hospital stay using a dedicated data sheet form. The principal investigator, statistician, and research fellow had access to the final datasets, while all participating researchers were permitted to view the final data. Data entry into the database was conducted systematically and monitored daily by a dedicated research fellow to ensure quality and accuracy.

### Procedure detail

All patients underwent median sternotomy. Prior to the initiation of cardiopulmonary bypass (CPB), each patient received a loading dose of heparin (3 mg/kg) to achieve an activated clotting time exceeding 480 seconds. Arterial and venous cannulations were performed according to the surgical procedure. During CPB, moderate hemodilution (hematocrit: 20%–26%) and systemic hypothermia (28°C–32°C) were employed. A roller pump with nonpulsatile flow rates of 2.0–2.4 L/m^2^/min and a membrane oxygenator were used. The activated clotting time was consistently maintained above 480 seconds, and mean arterial pressure was regulated between 50–70 mmHg. Cold blood cardioplegia was administered through an antegrade cardioplegia cannula inserted into the aortic root. At the conclusion of CPB, heparin was reversed with protamine sulfate (3.5 mg/kg).

**Fig. 1:**
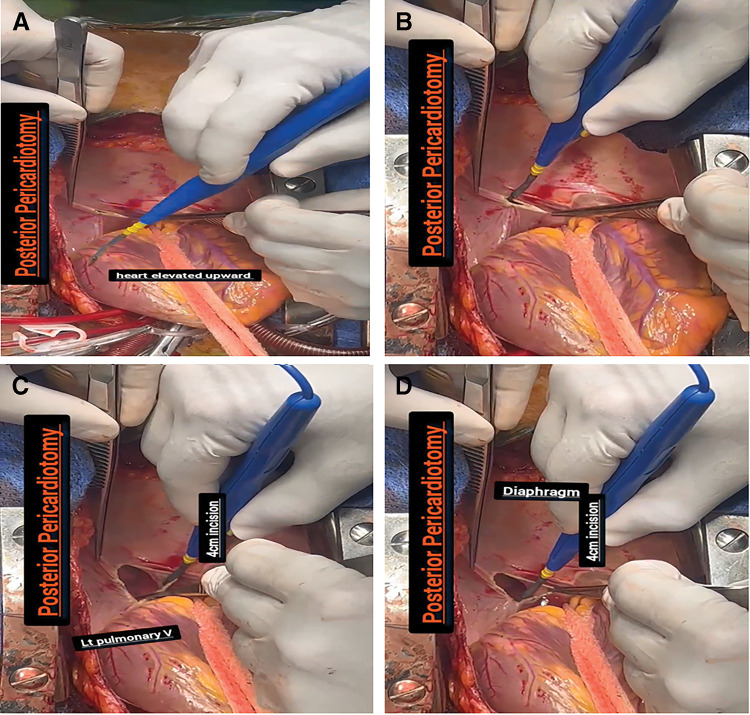
The posterior part of the pericardium following posterior pericardiotomy. (**A**) The heart has been lifted to expose the posterior pericardium. (**B**) A longitudinal incision was made parallel and posterior to the left phrenic nerve. (**C** and **D**) The incision was extended from the left inferior pulmonary vein to the diaphragm.

In the PP group, the PP technique was performed according to the type of cardiac procedure. In CABG surgery, PP was conducted following the completion of distal saphenous vein graft anastomosis and prior to the left internal mammary artery to the left anterior descending artery anastomosis. For valvular procedures or combined CABG and valvular surgeries, PP was performed after valve excision. A longitudinal incision measuring 4–5 cm was made parallel and posterior to the left phrenic nerve, extending from the left inferior pulmonary vein to near the diaphragm (**Fig. 1**),^14,15)^ any complications occurred during the creation of the PP has been recorded. Two chest tubes were placed at the conclusion of surgery for all patients, one (size 28 Fr) in the left pleural cavity and the other (size 32 Fr) in the anterior mediastinum. The left pleural drain was inserted into the pleural cavity and passed through a small intentional opening created in the left pleura (not through the PP opening); its tip was positioned in the lower left pleural space. This technique was applied uniformly in both groups to ensure standardization of pleural drainage. If the right side of the thorax was inadvertently opened, the anterior mediastinal drainage tube was passed through the right pleural opening before being positioned retrosternally. The anterior pericardium was left open in both groups. To avoid tube-induced ventricular arrhythmias, chest tube placement at the posterior aspect of the heart was avoided in both groups.

### Postoperative and outcomes

Following routine chest closure, postoperative management in the intensive care unit (ICU) involved frequent milking and stripping of the chest drains to ensure tube patency. Low-intermittent suction (−20 cmH_2_O) was applied. Blood drainage volumes were measured and recorded hourly. Chest tubes were removed on postoperative day 2 (POD 2) if drainage volume was less than 100 mL during the preceding 12 hours or if only serous drainage was observed. If these criteria were not met, reevaluation was conducted after 12 hours. All patients received routine postoperative antiarrhythmic prophylaxis with β-blockers unless contraindicated by bradycardia (heart rate <70 beats per minute), the need for epicardial pacing, the presence of an atrioventricular block, or concurrent administration of β-agonists. Systemic anticoagulation was initiated for postoperative atrial fibrillation (POAF) persisting longer than 24 hours or in cases of recurrent arrhythmic episodes. A standardized postoperative pain management protocol was applied to all patients. Postoperative imaging included an immediate chest radiograph (CXR) following surgery, with daily morning CXRs conducted throughout hospitalization.

The monitoring period spanned from the time of surgery to 30 days postoperatively. Patients discharged with moderate pleural effusion were scheduled for follow-up imaging 2–3 weeks post-discharge. The primary outcome included PE, cardiac tamponade, AF, and related complications. PE was defined as the accumulation of fluid in the pericardial space, assessed through transthoracic echocardiography (TTE). The presence of PE on 2-dimensional (2-D) TTE was evaluated using the criteria established by Bakhshandeh et al.^4)^. 2-D TTE assessments were conducted on PODs 1 and 5, and at 1 month post-surgery. The maximum diastolic separation between the pericardium and epicardium was measured at the tip of the mitral valve leaflet. Effusion classification was based on the diastolic echo-free space between the left ventricular posterior wall and the pericardium: <10 mm was classified as small, 10–20 mm as moderate, and >20 mm as large. In cases where moderate or large PE was identified, daily TTE assessments were performed to closely monitor effusion volume.

Primary outcomes in our study were postoperative PE and cardiac tamponade, and secondary outcomes included postoperative lounge of stay (LOS) in both the ICU and the hospital, hospital mortality, POAF, and re-admission.

### Statistical analysis

Statistical analysis was performed using IBM SPSS Statistics version 24.0 (IBM Corp., Armonk, NY, USA). Descriptive statistics were initially conducted, with categorical variables presented as frequencies and percentages, while continuous variables were summarized as means and standard deviations (SDs). The differences between the control and intervention groups were assessed using univariate analysis. Continuous variables were analyzed using either the Student' t-test or the Mann–Whitney U-test, depending on the data distribution. Categorical variables were evaluated using the chi-squared test, with corresponding 95% confidence intervals (CIs). A P-value of <0.05 was considered statistically significant. The risk factors for PE were adjusted in logistic, linear, and ordinal regression analyses for those variables with P <0.05 in univariate analysis. The baseline and procedural covariates were adjusted in logistic, linear, and ordinal regression multivariable analyses.

## Results

A total of 703 open cardiac surgeries were initially screened, and after applying the inclusion and exclusion criteria, 330 patients were enrolled in the final analysis of the current study. The study population comprised 212 male patients (64.2%) and 118 female patients (35.8%), with a mean age of 50.22 ± 14.7 years (range: 18–80 years).

### Baseline characteristics

The distribution of comorbidities and risk factors among the study population, categorized based on the PP group (Group I) and the nonintervention group (Group II), is presented in **Table 1**. Notably, the nonintervention group exhibited a higher left ventricular ejection fraction (LVEF) (%) (56 vs. 52; P = 0.049) compared to the intervention group. There were no significant differences between the 2 groups in terms of age, sex distribution, and risk factor (all P >0.05). To address potential confounding factors, a multivariate logistic regression analysis was conducted to adjust for baseline imbalances in LVEF and operative/CPB times.

**Table 1:** Baseline comorbidity and risk factors in both study groups

Variables	Total (n = 330)	PP group (n = 165)	Control group (n = 165)	P-value
Hypertension, n (%)	114 (34.5)	60 (36.4)	54 (32.7)	0.487
Diabetes mellitus, n (%)	91 (27.6)	50 (30.3)	41 (24.8)	0.268
Smoker, n (%)	72 (21.8)	36 (21.8)	36 (21.8)	1.000
COPD, n (%)	19 (5.8)	8 (4.8)	11 (6.7)	0.487
PAD, n (%)	7 (2.1)	4 (2.4)	3 (1.8)	0.702
MI, n (%)	154 (46.7)	82 (49.7)	72 (43.6)	0.270
Stroke or TIA, n (%)	32 (9.7)	19 (11.5)	14 (8.5)	0.457
Hematocrit, %	38.0 (35.0–40.0)	38.0 (35.0–40.0)	38.6 (35.0–40.7)	0.298
Creatinine, mg/dl	0.9 (0.7–1.0)	0.9 (0.7–1.0)	0.9 (0.75–1.0)	0.251
LVEF, %	53 (45–62)	52 (45–60)	56 (48–64)	0.049
Left atrial diameter, cm	4.2 (3.6–5.2)	4.2 (3.9–5.1)	4.2 (3.5–5.3)	0.474
CHA 2DS2-VASc score	2 (1–3)	2 (1–2)	2 (1–2.5)	0.003
NYHA I–II, n (%)	136 (41.2%)	71 (43.0)	65 (39.4)	0.531
NYHA III–IV, n (%)	158 (47.9%)	79 (47.9)	79 (47.9)	0.531

Data presented as median (IQR), and number (n) with (%).

PP: posterior pericardiotomy; COPD: chronic obstructive pulmonary disease; PAD: peripheral arterial disease; MI: myocardial infarction; TIA: transient ischemic attack; LVEF%: left ventricular ejection fraction; NYHA: New York Heart Association class; IQR: interquartile range

### Operative variables

The median CPB time (97 vs. 111 minutes; P <0.001) and operation duration (200 vs. 172 minutes; P <0.001) in the nonintervention group were longer than in the intervention group (**Table 2**). The difference in CPB time and operation duration between groups is attributed to variations in surgical complexity and team experience. To account for this, these variables were included in the multivariate analysis.

**Table 2:** Operative variables among patients in both study groups

Variables	Total (n = 330)	PP group (n = 165)	Control group (n = 165)	P-value
**Type of surgery**				
CABG, n (%)	175 (53.0)	87 (52.7)	88 (53.3)	0.451
Valve replacement, n (%)	138 (41.8)	67 (40.6)	71 (43.0)	0.451
Combined, n (%)	17 (5.2)	11 (6.7)	6 (3.6)	0.451
Number of grafts, mean ± SD	3.2 ± 0.7	3.3 ± 0.7	3.1 ± 0.78	0.146
Cross-clamp time, min	70 (55.0–94.0)	69 (55–90)	75 (55.0–100.3)	0.183
Cardiopulmonary bypass time, min	104 (87.0–130.0)	97 (80–120)	111 (94.0–142.0)	0.000
Operation duration, min	184 (160.0–130.0)	172 (150.0–195.0)	200 (180.0–240.0)	0.000

Data are presented as median (IQR) and number (n) with (%).

PP: posterior pericardiotomy; CABG: coronary artery bypass grafting; SD: standard deviation

### Primary outcomes and related complications

In the present study, postoperative PE was observed in 116 patients (35.2%). The incidence of both early and late PE was significantly lower in the PP group compared to the nonintervention group (19.4% vs. 44.8%; P = 0.001). When stratified by the type of surgical procedure, the incidence of PE among CABG patients was significantly lower in the PP group compared to the nonintervention group (18.4% vs. 81.6%; P = 0.001). Similarly, for isolated valve surgeries, the incidence of PE was also lower in the PP group (36% vs. 64.7%; P = 0.001). However, the incidence of postoperative left pleural effusion was not significantly higher in the PP group compared to the nonintervention group (26.7% vs. 19.4%; P = 0.117).

Pleural effusion was detected in 100 patients (30.3%) in the study cohort, with 52 patients (31.5%) in the pericardiotomy group and 48 patients (29.1%) in the nonintervention group. There was no statistically significant difference between the 2 groups regarding the incidence of pleural effusion or the requirement for pleural drainage. The incidence of postoperative left pleural effusion was 44 patients (26.7%) in the posterior left pericardiotomy group and 32 patients (19.4%) in the nonintervention group. The drain duration for left pleural tubes was similar between groups (median: 2 days), and no significant difference in pleural infections was found. With respect to postoperative pleural drainage, 8 out of 44 patients (18.2%) in the pericardiotomy group and 6 out of 32 patients (18.7%) in the nonintervention group required intervention. This difference was not statistically significant (P = 0.117).

PE cases were classified based on the extent of pericardial space expansion: small (<10 mm) in 65 patients (56% of cases with effusion), moderate (10–20 mm) in 27 patients (23.2%), and large (>20 mm) in 24 patients (20.8%). Patients with small PE were treated with diuretics and anti-inflammatory drugs for 2 weeks, and no further intervention was required. Follow-up echocardiography performed during the 1st postoperative month confirmed the complete resolution of PE in all cases. Among the 27 patients (23.2%) with moderate PE, treatment with diuretics and anti-inflammatory drugs was extended for 2–4 weeks. Of these, only 2 patients (7.4%) developed pericardial tamponade, necessitating intervention. Follow-up echocardiography at 1 month postoperatively identified mild residual PE in 3 cases.

Clinical manifestations of cardiac tamponade, including hypotension, tachycardia, and decreased urine output, were observed in 23 patients (20%) with PE. Among patients with progressive large PE, 21 out of 24 developed cardiac tamponades, necessitating emergency intervention. Management included subxiphoid pericardiotomy in 6 patients and emergent resternotomy in 15 patients. Tamponade occurred early (<10 days postoperatively) in 6 patients, whereas late-onset tamponade (days 14–23) was documented in 9 patients. The incidence of cardiac tamponade was significantly higher following valve replacement (17 patients) compared to CABG (5 patients) or combined surgical procedures (1 patient); P <0.05. Furthermore, cardiac tamponade was more prevalent in the nonintervention group compared to the PP group (11.5% vs. 2.4%; P = 0.001).

POAF was observed in 49 patients (14.8%), with 17 patients (10.3%) in the PP group and 32 patients (19.4%) in the nonintervention group. The incidence of POAF was significantly higher among patients with PE (30.2% vs. 6.5%; P <0.001) compared to patients without PE. The occurrence of POAF was significantly less frequent in the pericardiotomy group (10.7% vs. 19.4%; P = 0.02) than in the nonintervention group (**Table 3**).

**Table 3:** Primary outcome of patients in both study groups

Variables	Total (n = 330)	PP group (n = 165)	Control group (n = 165)	P-value
Pericardial effusion	116 (35.2%)	34 (20.6%)	82 (49.7%)	<0.001
Early pericardial effusion	106 (32.1%)	32 (19.4%)	74 (44.8%)	<0.001
Late pericardial effusion	35 (10.6%)	7 (4.2%)	28 (17.0%)	<0.001
Pleural effusion	100 (30.3%)	52 (31.5%)	48 (29.1%)	0.632
Left pleural effusion	76 (23%)	44 (26.7%)	32 (19.4%)	0.117
Cardiac tamponade	23 (7%)	4 (2.4%)	19 (11.5%)	0.001
POAF	49 (14.8%)	17 (10.3%)	32 (19.4%)	0.020

Data are n (%).

POAF: postoperative atrial fibrillation; PP: posterior pericardiotomy

### Secondary outcomes

The medians total ventilation time [3.0 (3.0–4.0) vs. 4.0 (3.0–6.0) hr; P <0.001] and ICU stay [23.0 (22.0–26.0) vs. 24.0 (23.0–48.0) hr; P <0.001] were shorter in pericardiotomy group than in the nonintervention group. Moreover, the median of LOS in hospital was shorter [6.0 days (5.0–7.0) vs. 6.0 days (6.0–9.0); P = 0.002] in the intervention group compared to the nonintervention group. The overall hospital mortality rate in this study was 2.1% of patients, and there was no statistical difference between the 2 groups (P = 0.052). Causes of in-hospital mortality included septic shock (n = 2), myocardial infarction (n = 1), stroke (n = 1), and cardiac tamponade (n = 3, all in the control group). Hospital readmission within 30 days after discharge occurred in 43 (13%) patients, and there was no statistically significant difference between the 2 groups (P = 0.141) (**Table 4**).

**Table 4:** Postoperative variables in our patients (n = 330)

Variables	Total (n = 330)	PP group (n = 165)	Control group (n = 165)	P-value
Total ventilation, time/hr	3.5 (3.0–5.0)	3.0 (3.0–4.0)	4.0 (3.0–6.0)	0.000
Total ICU, time/hr	24.0 (22.0–41.0)	23.0 (22.0–26.0)	24.0 (23.0–48.0)	0.000
Need IABP	4 (1.2%)	2 (1.2%)	2 (1.2%)	1.000
Hospital stays, days median (IQR)	6.0 (5.0–8.0)	6.0 (5.0–7.0)	6.0 (6.0–9.0)	0.002
Hospital mortality, n (%)	7 (2.1)	1 (0.6)	6 (3.6)	0.052
30-Day re-admission, n (%)	43 (12.5)	17 (10.3)	26 (17.3)	0.141

IABP: intra-aortic balloon pump; PP: posterior pericardiotomy; ICU: intensive care unit; IQR: interquartile range

### Multivariable regression summary

PP was an independent protective factor (odds ratio [OR] = 0.223, 95% CI: 0.122–0.407; P <0.001). Smoking was associated with increased risk (OR = 2.147; P = 0.033). Other variables, including EF, CPB time, and operation duration, were not statistically significant (**Table 5**).

**Table 5:** Multivariable logistic regression analysis of baseline and procedural covariates

Variable	Odd ratio	95% Confidence interval	P-value
Age, years	1.008	0.985–1.032	0.507
Gender	1.344	0.738–2.449	0.334
Hypertension	1.104	0.552–2.207	0.780
Diabetes mellitus	1.273	0.600–2.202	0.529
Smoker (current/recently)	2.148	1.062–4.344	0.033
Previous myocardial infarction	1.516	0.743–3.094	0.252
Left atrium size, cm	0.971	0.725–1.299	0.841
Preoperative hematocrit, %	0.977	0.920–3.199	0.451
Preoperative creatinine	1.340	0.562–3.199	0.509
Left ventricular ejection fraction, %	1.045	0.971–1.125	0.240
Cross-clamp time, min	0.989	0.974–1.005	0.180
Cardiopulmonary bypass time, min	1.011	0.991–1.031	0.286
Operation duration, min	1.008	0.994–1.021	0.265
Posterior pericardiotomy	0.223	0.122–0.407	0.001

## Discussion

This randomized trial is the 1st to evaluate the effectiveness of pericardiotomy in reducing PE, POAF, and related complications in Yemen. The findings demonstrate that performing PP at the time of cardiac surgery significantly reduces the incidence of POAF, PE, and cardiac tamponade. These results are consistent with previous meta-analyses, randomized controlled trials, and the majority of retrospective cohort studies.^16–18)^

The mean patient age was relatively young (50 years) compared to older cohorts commonly seen in Western cardiac centers,^19–21)^ possibly reflecting regional demographic differences. In our setting, this early onset of cardiac disease is likely multifactorial, influenced by poor nutritional habits, sedentary lifestyle, widespread smoking, lack of structured physical activity, and absence of regular health screening programs. Environmental stress, limited access to primary prevention services, and a high prevalence of untreated hypertension and diabetes may also contribute to the earlier manifestation of cardiovascular disease

Postoperative PE is a common complication following cardiac surgery, with incidence rates ranging from 4.7% to 85%, depending on the diagnostic modality utilized. PE is often localized in the posterior pericardial space and is associated with increased morbidity and mortality.^22)^ Effective drainage, appropriate placement, and maintenance of surgical drain patency are critical for ensuring optimal postoperative outcomes.^23)^ Moreover, adopting a semi-sitting position in intensive care settings has been shown to enhance chest drainage during the early postoperative period.^3)^

PP acts as a protective mechanism, serving as a safety valve that reduces the risk of cardiac tamponade and both early and late PE.^24)^ Several studies have demonstrated the benefits of PP in minimizing postoperative PE.^15,25–28)^ Consistent with these findings, our study revealed that PP significantly decreased the incidence of PE following cardiac surgery, with both early and late effusions being notably less frequent in the PP group compared to the control group. Similar results were observed in the PALACS trial,^26)^ which reported a significantly lower incidence of postoperative PE in the PP group (12% vs. 21%; relative risk 0.58, 95% CI: 0.37–0.91). A meta-analysis by Soletti et al.^15)^ further substantiated these findings, demonstrating a reduced incidence of PE in the PP group. Additionally, multiple studies have corroborated these results, confirming that PP is an effective technique for reducing postoperative PE compared to conventional surgical approaches.^3,8,16,23,25,28–30)^

Cardiac tamponade is a life-threatening complication following cardiac surgery. In the present study, the incidence of cardiac tamponade was significantly lower in the PP group compared to the nonintervention group. Several clinical studies and meta-analyses have assessed the efficacy of PP in reducing the risk of cardiac tamponade.^31)^ Soletti et al.^15)^ conducted a meta-analysis demonstrating a significantly lower risk of cardiac tamponade in patients who underwent PP compared to the nonintervention group. Similarly, Abdelaziz et al.,^28)^ in a systematic review and meta-analysis, found that PP significantly reduced the incidence of postoperative cardiac tamponade. However, some studies, such as the PALACS trial, reported no significant difference in the incidence of cardiac tamponade between the PP and nonintervention groups.^26)^ Some patients in our study were discharged from the hospital after valve surgery with small, clinically insignificant PEs but were later readmitted due to tamponade approximately 1–2 weeks postoperatively. It is crucial to closely monitor these patients after discharge and educate both patients and their relatives about potential nonspecific symptoms. This finding is consistent with results that have been reported in other studies.^32)^

In our clinical trial, the PP significantly reduced the incidence of POAF. The most recent large-scale meta-analysis conducted by Abdelaziz et al.^28)^ included 25 trials with a total of 4467 patients; 22 studies encompassing 4300 patients reported POAF outcomes. The cumulative incidence of POAF was significantly lower (11.7% vs. 23.67%) in the PP group than in the control group, with pooled OR and 95% CI for POAF of 0.49 (95% CI: 0.38–0.61), further supporting the efficacy of PP in reducing POAF. Similarly, Soletti et al.^15)^ and Gaudino et al.^26)^ also reported a significantly lower incidence of AF in the PP group. In contrast, Arbatli et al.,^33)^ Cakalagaoglu et al.,^34)^ and Asimakopoulos et al.^21)^ demonstrated that PP was more effective in draining the PE, but the incidence of POAF was not significantly reduced compared with the control group.

In our study, we found that patients who underwent PP had a significantly shorter postoperative in-hospital stay compared to the nonintervention group. This result is consistent with findings reported by Arsan et al.,^35)^ and Ekim et al.^6)^. The factors contributing to a shorter hospital stay can be explained by a lower incidence of pericardial and pleural effusions, cardiac tamponade, and re-exploration requiring drainage procedures in the PP group. Moreover, the reduction of POAF in the PP group, which is a significant cause of prolonged hospital stay, may also contribute to this outcome.

No PP-related complications were observed in our study. Specifically, there were no instances of esophageal, aortic, diaphragmatic, or lung injury, nor any phrenic nerve palsy. However, surgeons should be aware of rare risks such as ventricular arrhythmias, graft kinking, or posterior wall injury, as documented in prior studies. Yorgancioǧlu et al.^36)^ have previously reported hemodynamic instability and uncontrollable arrhythmias due to protrusion of a sequential graft through the PP opening. In summary, we found that performing PP at the time of surgery was associated with a significant reduction in the incidence of POAF in patients undergoing cardiac operations. A confirmatory multicenter trial including the entire spectrum of cardiac surgery operations is needed to quantify the potential clinical benefits of the intervention in cardiac surgery patients.

### Limitation

This study has several limitations. First, it was conducted at a single center, which may limit the generalizability of the results to other institutions with different surgical practices and patient populations. Second, the study follow-up was limited to 30 days postoperatively, which may not capture late-onset PE or arrhythmias. Third, the relatively young mean age of the study population may not reflect the demographic profile of cardiac surgery patients in Western settings. Finally, although multivariable regression was used to adjust for confounders, residual confounding due to unmeasured variables cannot be completely ruled out.

## Conclusion

PP is a simple and safe method that significantly reduces the incidence of PE and cardiac tamponade after cardiac surgery by improving pericardial drainage. Further multicenter studies are warranted to provide more robust results.

## Acknowledgments

We thank our statistician Dr. Mohamed Althurasi for conducting the multivariate analysis.

## Declarations

### Ethics approval and consent to participate

Each subject provided signed informed consent; the study was performed considering the Declaration of Helsinki and was approved by the Institutional Review Committee of Taiz University, Taiz, Yemen.

### Consent for publication

Not applicable.

### Funding

There was no funding source for this study.

### Disclosure statement

All authors have no disclosures to report or conflict of interest.

### Data availability

The data that support the findings of this study are available from the corresponding author upon reasonable request.

### Authors’ contributions

Ismail Al Shameri: data collection, management, and analysis, and writing the major part of the manuscript. Abudar A. Al-ganadi: discussion and review of the manuscript. Tarq Noman: review of the results section of the manuscript, Mahdi A. Kadry: review of the abstract and conclusion of the manuscript. Ihab M. Elsharkawy: review of the introduction of the manuscript. Naseem Al-wsabi: preparation of tables and figure. Ayman A. Mohammed: preparation of the manuscript. All authors have read and approved the final version of the manuscript.
